# Singular Value Decomposition (SVD) Method for LiDAR and Camera Sensor Fusion and Pattern Matching Algorithm

**DOI:** 10.3390/s25133876

**Published:** 2025-06-21

**Authors:** Kaiqiao Tian, Meiqi Song, Ka C. Cheok, Micho Radovnikovich, Kazuyuki Kobayashi, Changqing Cai

**Affiliations:** 1 Electrical and Computer Engineering, Oakland University, Rochester, MI 48309, USA; tian2@oakland.edu (K.T.); cheok@oakland.edu (K.C.C.); mtradovn@oakland.edu (M.R.); 2 Department of Mathematics and Statistics, Oakland University, Rochester, MI 48309, USA; msong@oakland.edu; 3 Department of Advanced Sciences, Hosei University, Tokyo 184-8584, Japan; ikko@hosei.ac.jp; 4 College of Electrical and Information Engineering, Changchun Institute of Technology, 395 Kuan Ping Road, Changchun 130103, China

**Keywords:** LiDAR and camera data sensor fusion, singular value decomposition, gradient descent, pattern matching, error detection

## Abstract

LiDAR and camera sensors are widely utilized in autonomous vehicles (AVs) and robotics due to their complementary sensing capabilities—LiDAR provides precise depth information, while cameras capture rich visual context. However, effective multi-sensor fusion remains challenging due to discrepancies in resolution, data format, and viewpoint. In this paper, we propose a robust pattern matching algorithm that leverages singular value decomposition (SVD) and gradient descent (GD) to align geometric features—such as object contours and convex hulls—across LiDAR and camera modalities. Unlike traditional calibration methods that require manual targets, our approach is targetless, extracting matched patterns from projected LiDAR point clouds and 2D image segments. The algorithm computes the optimal transformation matrix between sensors, correcting misalignments in rotation, translation, and scale. Experimental results on a vehicle-mounted sensing platform demonstrate an alignment accuracy improvement of up to 85%, with the final projection error reduced to less than 1 pixel. This pattern-based SVD-GD framework offers a practical solution for maintaining reliable cross-sensor alignment under calibration drift, enabling real-time perception systems to operate robustly without recalibration. This method provides a practical solution for maintaining reliable sensor fusion in autonomous driving applications subject to long-term calibration drift.

## 1. Introduction

Sensor fusion is a fundamental technique in robotics and autonomous systems that combines data from multiple heterogeneous sensors to achieve a more comprehensive and accurate perception of the surrounding environment. Among various sensor combinations, the fusion of Light Detection and Ranging (LiDAR) and camera data has gained significant attention due to their complementary characteristics—LiDAR provides precise geometric and depth information, while cameras capture rich semantic and visual context. This multimodal fusion enhances environmental understanding, leading to improved object detection, scene interpretation, and overall system reliability, which are crucial for Advanced Driver Assistance Systems (ADAS) and autonomous navigation [1,2,3,4].

Numerous fusion strategies have been developed to optimize the integration of LiDAR and camera data, including fully convolutional neural networks [2], deep fusion architectures [5], and segmentation-based approaches [6,7,8]. These algorithms aim to address challenges in data alignment, improve robustness to noise, and ensure real-time performance in dynamic environments. However, the effectiveness of such methods critically depends on the accuracy of sensor calibration and the process of determining the spatial and temporal relationships between sensors.

Traditional calibration techniques often rely on manual procedures using checkerboards or custom-designed targets [9,10,11,12,13]. While these methods can achieve high precision in controlled environments, they require repetitive labor, are sensitive to installation errors, and are impractical for large-scale or long-term deployments. Moreover, calibration parameters are prone to drift over time due to environmental factors such as temperature variation, vibration, and sensor wear, degrading fusion performance unless periodic recalibration is performed [14].

To address these limitations, recent research has explored targetless calibration methods that extract features from natural scenes—such as edges, planes, or point correspondences—to estimate the extrinsic transformation between sensors [14,15,16]. However, these methods can still struggle under severe noise, occlusion, or perspective distortion. Pattern matching-based approaches represent a promising alternative by aligning geometric structures such as contours, convex hulls, or projected keypoints between modalities. Some studies apply mutual information or optimization-based matching [15], while others incorporate learned correspondences through deep models [16].

Singular value decomposition (SVD)-based methods have emerged as a mathematically robust and interpretable tool for estimating rigid transformations from point sets. The Kabsch algorithm, based on SVD, has been widely adopted in registration and poses estimation tasks due to its closed-form optimality under least-squares errors [17,18,19,20]. When combined with robust feature extraction, this technique offers a powerful framework for aligning heterogeneous sensor data.

In this paper, we introduce a novel pattern matching algorithm for LiDAR–camera fusion that leverages the strengths of SVD and iterative refinement through gradient descent. Rather than directly re-estimating intrinsic or extrinsic parameters, our method aligns projected 3D point cloud features with 2D image contours by matching spatial patterns. This strategy allows us to correct sensor misalignment without requiring calibration targets, enabling efficient, repeatable fusion correction in real-world autonomous systems. Experimental results demonstrate that our method significantly improves alignment under varying environmental and calibration drift conditions.

## 2. Methodology

### 2.1. Sensor Setup and Calibration

**Figure 1:**
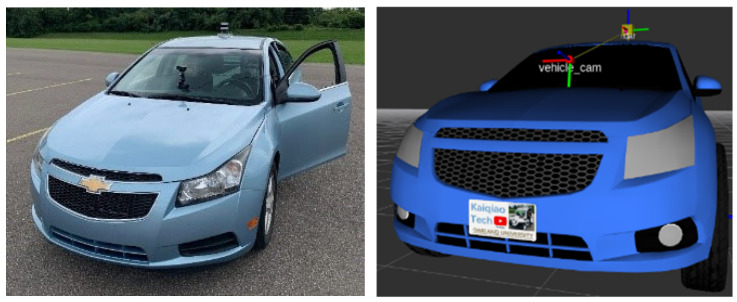
Vehicle platform and ROS simulation.

For development and testing, we employ a real-world sensing platform consisting of a Chevrolet (Detroit, MI, USA) Cruise vehicle equipped with an Ouster OS1-64 LiDAR (Ouster, Inc., San Francisco, CA, USA) mounted on the roof and a Logitech RGB camera (Logitech, Lausanne, Switzerland) positioned on the windshield (see Figure 1). To facilitate algorithm evaluation and simulation under controlled conditions, a simulation of the vehicle was constructed in the Robot Operating System (ROS) environment (ROS Noetic, Ubuntu 20.04). This virtual model preserves the real vehicle’s geometry and sensor placement, enabling reliable reproduction of sensor data streams and frame transformations.

**Figure 2:**
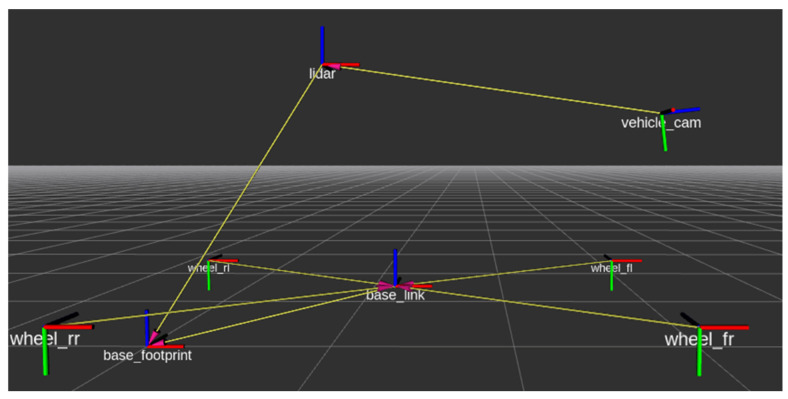
The vehicle and sensor system transfer frame tree.

As illustrated in Figure 2, the transform (TF) tree of the vehicle model defines the spatial relationships between the vehicle body and its mounted sensors using six degrees of freedom—roll, pitch, yaw, and x, y, z translations. The base_footprint frame represents the vehicle’s reference point on the ground, typically defined at the geometric center between the rear wheels. The vehicle_cam frame, corresponding to the camera’s physical location, is defined as a child frame relative to the LiDAR frame.

**Figure 3:**
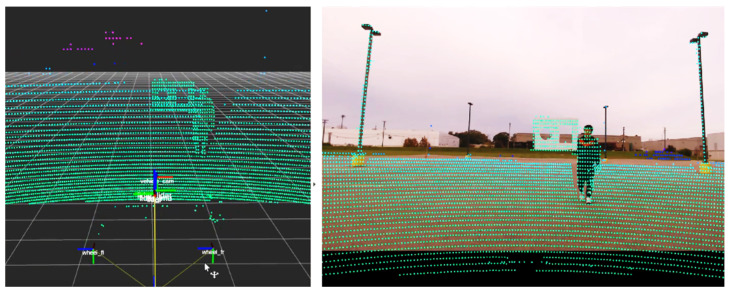
LiDAR and camera manual calibration. The left image shows the LiDAR point cloud in the 3D coordinate frame, while the right image displays the LiDAR points (green dots) projected onto the camera image.

As illustrated in Figure 3, a checkerboard calibration target with two cut-out holes was used to evaluate the alignment between LiDAR and camera data. Within the ROS RViz environment, both the LiDAR point cloud and the vehicle’s TF tree are visualized, demonstrating the spatial consistency between the 3D LiDAR data and the 2D image plane. The resulting point cloud captures distinct environmental features such as the ground surface, vertical light poles, and the calibration board itself, verifying the accuracy of the fusion setup.

**Figure 4:**
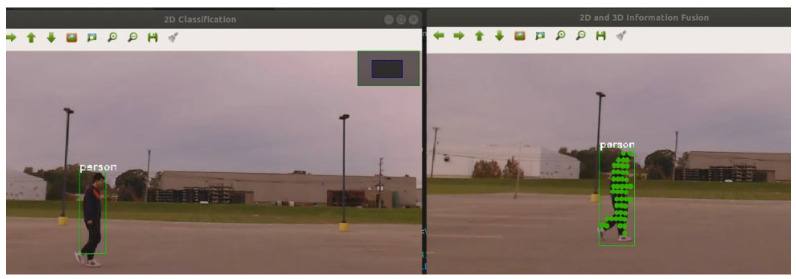
Camera CNN detection result with LiDAR point cloud projected onto the 2D image.

To project LiDAR point cloud data onto the 2D image plane, we apply a standard extrinsic and intrinsic calibration procedure. The camera intrinsics (focal length, principal point, and distortion coefficients) and the extrinsic transformation between the LiDAR and camera coordinate frames $\left[R|t\right]$ were determined using a checkerboard target and validated in ROS. This transformation allows 3D LiDAR points (x,y, z), to be mapped onto 2D image coordinates (u, v) using the following projection model:

(1) $$P=K\times \left[R|t\right]$$

where K is the camera intrinsic matrix. This projected mapping enables spatial comparison between LiDAR and camera features, which is essential for the proposed SVD-based pattern matching algorithm. We employed the YOLOv5s architecture for object detection, consisting of CSPDarknet53 backbone, PANet neck, and YOLO head layers. The model was pretrained on the COCO dataset (80 object categories, 118 k images) and fine-tuned on a custom dataset of 3000 manually labeled pedestrian images captured under urban driving conditions. The input resolution was 640 × 480 pixels, using a stochastic gradient descent optimizer with a learning rate of 0.01 and batch size of 16. The confidence threshold for detection was set to 0.4 during inference. As shown in Figure 4, the CNN detection result from the camera is overlaid with the projected LiDAR point cloud on the 2D image.

### 2.2. Pattern Matching Algorithm

**Figure 5:**
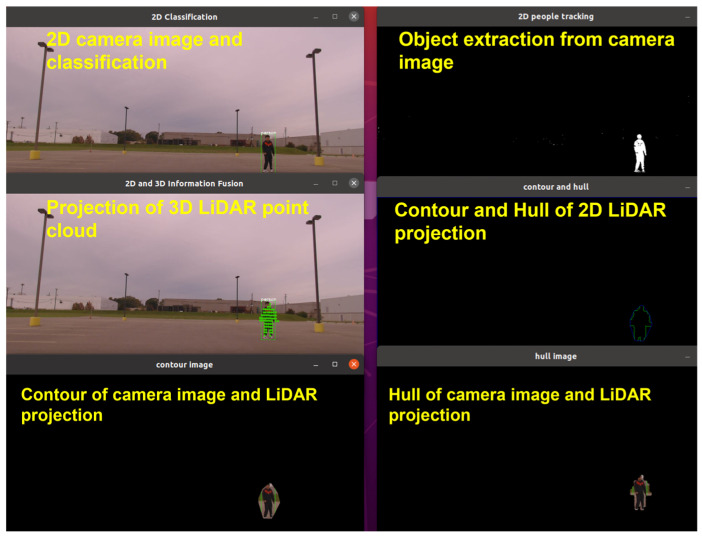
Processing pipeline of the proposed pattern matching algorithm. Top-left: 2D camera image with classification results. Middle-left: projection of 3D LiDAR point cloud onto the image. Bottom-left: overlaid contours of the camera image and LiDAR projection. Top-right: object extraction from the camera image. Middle-right: contour and convex hull of 2D LiDAR projection. Bottom-right: convex hulls from both camera and LiDAR data.

The proposed algorithm projects LiDAR point clouds onto the camera image plane, followed by the extraction of object contours and convex hulls from both modalities to capture their geometric features. Singular value decomposition (SVD) is applied to estimate the initial transformation parameters, including rotation, translation, and scale, that align the two datasets. This estimate is further refined through gradient descent (GD) optimization, which minimizes a cost function based on the residual geometric discrepancy. The combined SVD-GD approach enables robust alignment correction under varying degrees of calibration drift and sensor misalignment. Figure 5 illustrates the data preprocessing pipeline, including CNN-based object detection, LiDAR projection onto the image plane, and contour and convex hull extraction from both modalities. The extracted features are then used for SVD-based alignment and subsequent GD refinement.

The proposed pattern matching algorithm identifies and corrects sensor misalignment by minimizing discrepancies between the extracted geometric features, leading to improved fusion accuracy. By applying this algorithm, the discrepancies between the two datasets can be minimized, leading to more accurate and precise sensor fusion results.

Let the respective LiDAR and camera data at frame i be denoted as matrices $p_{i}$ and $q_{i}$, respectively, where each matrix contains the spatial features or point correspondences captured by each sensor at that frame:

(2) $$p_{i}={\left[\begin{array}{c} p_{x}\\ p_{y}\\ p_{z} \end{array}\right]}_{i}=\left[\begin{array}{c} p_{x_{i}}\\ p_{y_{i}}\\ p_{z_{i}} \end{array}\right]\;\;\;\;\;\;\;\;\;\;\;\;\;\;\;q_{i}={\left[\begin{array}{c} q_{x}\\ q_{y}\\ q_{z} \end{array}\right]}_{i}=\left[\begin{array}{c} q_{x_{i}}\\ q_{y_{i}}\\ q_{z_{i}} \end{array}\right]$$

With reference to the *p*-frame, the relative transformation from the *p*-frame to the *q*-frame can be represented by a translation vector $d_{q}^{p}$ and a rotation matrix $R_{q}^{p}$, where

(3) $$\begin{array}{c} d_{q}^{p}={\left[\begin{array}{c} x\\ y\\ z \end{array}\right]}_{q}^{p}\\ R_{q}^{p}=\left[\begin{array}{ccc} cos\psi & -sin\psi & 0\\ sin\psi & cos\psi & 0\\ 0 & 0 & 1 \end{array}\right]\;\left[\begin{array}{ccc} cos\theta & 0 & sin\theta \\ 0 & 1 & 0\\ -sin\theta & 0 & cos\theta \end{array}\right]\left[\begin{array}{ccc} 1 & 0 & 0\\ 0 & cos\phi & -sin\phi \\ 0 & sin\phi & cos\phi \end{array}\right]\\ =\left[\begin{array}{ccc} cos\psi cos\theta & -sin\psi cos\theta +cos\psi sin\theta sin\phi & sin\psi sin\phi +cos\psi sin\theta cos\phi \\ sin\psi cos\theta & cos\psi cos\phi +sin\psi sin\theta sin\phi & -cos\psi sin\phi +sin\psi sin\theta cos\phi \\ -sin\theta & cos\theta sin\phi & cos\theta cos\phi \end{array}\right] \end{array}$$

The forward kinematics relationship between the position vector p and q is given by

(4) $$p_{i}=d_{q}^{p}+R_{q}^{p}q_{i}$$

If a collection of m data vectors is obtained at each frame, the dataset can be represented as a matrix, where each column (or row) corresponds to a single data vector captured at a specific frame.

(5) $$P=\left[p_{1}\;p_{2}\ldots \;p_{m}\right]\;\;\;\;\;\;\;\;\;\;\;\;\;\;\;\;\;\;\;Q=\left[q_{1}\;q_{2}\ldots \;q_{m}\right]$$

Then, the matrix relationship is as follows:

(6) $$P=D_{q}^{p}+R_{q}^{p}Q\;\;\;\;\;\;\;\;\;\;\;\;\;\;\;\;\;\;\;\;\;\;D_{q}^{p}=d_{q}^{p}1_{1\times m}$$

The relationship between the means of the data is similarly expressed as

(7) $$\bar{p}=d_{q}^{p}+R_{q}^{p}\bar{p}\;where,\;\;\;\;\;\bar{p}=\frac{1}{m}\sum_{i=1}^{m}p_{i},\;\bar{q}=\frac{1}{m}\sum_{i=1}^{m}q_{i}$$

Let the matrix H be defined as the product of the centered data matrices:

(8) $$H=\left[P-\bar{P}\right]{\left[Q-\bar{Q\;}\right]}^{\prime }$$

where $\left[P-\bar{P}\right]$ represents the deviation of the LiDAR data matrix P from its mean $\bar{P}=\bar{p}1_{1\times m}$ and ${\left[Q-\bar{Q\;}\right]}^{\prime }$ represents the transpose of the deviation of the camera data matrix Q from its mean $\bar{Q}=\bar{q}1_{1\times m}$.

To extract the rotational relationship between the two sets of observations, we apply singular value decomposition (SVD) to H, yielding

(9) $$H=USV^{\prime }$$

where U and V are unitary matrices in the minimal singular value decomposition, and S is a diagonal matrix of singular values of H. This decomposition allows for the estimation of the optimal rotation matrix that aligns the LiDAR and camera data in a least-squares context.

Use forward kinematics to express H as

(10) $$H=\left[D_{q}^{p}+R_{q}^{p}Q-D_{q}^{p}+R_{q}^{p}\bar{Q}\right]{\left[Q-\bar{Q\;}\right]}^{\prime }\;=R_{q}^{p}\left[Q-\bar{Q\;}\right]{\left[Q-\bar{Q\;}\right]}^{\prime }$$

Use a similarity transformation to decompose the symmetric matrix, yielding the following relationship:

(11) $$\begin{array}{c} H=R_{q}^{p}T_{QQ}\Lambda_{QQ}T_{QQ}^{\prime }\\ \Lambda_{QQ}=\left[diag\left\{\lambda_{j}\left(\left[Q-\bar{Q\;}\right]{\left[Q-\bar{Q\;}\right]}^{\prime }\;\right),\;j=1,2,3;\lambda_{1}\geq \lambda_{2}\geq \lambda_{3}\;\right\}\right]\\ \lambda_{j}=eigenvalue\;of\;\left[Q-\bar{Q\;}\right]{\left[Q-\bar{Q\;}\right]}^{\prime }\\ T_{QQ}=modal\;matrix\;of\;\left[Q-\bar{Q\;}\right]{\left[Q-\bar{Q\;}\right]}^{\prime }\\ {\;\;T}_{QQ}^{\prime }=T_{QQ}\;\;or\;\;T_{QQ}^{\prime }=I \end{array}$$

We decompose H using the SVD algorithm:

(12) $$H=USV^{\prime }$$

(13) $$Where,\;\;\;\;\;\;\;\;S=\left[diag\left\{\sqrt{\lambda_{j}(H^{\prime }H)},\;j=1,2,3;\lambda_{1}\geq \lambda_{2}\geq \lambda_{3}\;\right\}\right]\;UU^{\prime }=U^{\prime }U=I\;V^{\prime }V={VV}^{\prime }=I$$

It can be observed that

(14) $$H^{\prime }H=\left[Q-\bar{Q\;}\right]{\left[Q-\bar{Q\;}\right]}^{\prime }{R_{q}^{p}}^{\prime }R_{q}^{p}\left[Q-\bar{Q\;}\right]{\left[Q-\bar{Q\;}\right]}^{\prime }\;={\left[\left[Q-\bar{Q\;}\right]{\left[Q-\bar{Q\;}\right]}^{\prime }\right]}^{2}$$

As a result, we find that

(15) $$S=\left[diag\left\{\sqrt{\lambda_{j}\left(H^{\prime }H\right)},\;j=1,2,3\;\right\}\right]\;=\left[diag\left\{\sqrt{\lambda_{j}\left({\left[\left[Q-\bar{Q\;}\right]{\left[Q-\bar{Q\;}\right]}^{\prime }\right]}^{2}\right)},\;j=1,2,3\;\right\}\right]\;=\left[diag\left\{\lambda_{j}\left(\left[Q-\bar{Q\;}\right]{\left[Q-\bar{Q\;}\right]}^{\prime }\;\right),\;j=1,2,3\;\right\}\right]=\Lambda_{QQ}$$

From the relationship $H=R_{q}^{p}T_{QQ}\Lambda_{QQ}T_{QQ}^{\prime }=USV^{\prime }$, one finds that

(16) $$H=R_{q}^{p}T_{QQ}\;V^{\prime }=T_{QQ}^{\prime }$$

Accordingly, the rotation matrix that aligns the LiDAR coordinate frame with the camera coordinate frame is given by:

(17) $$R_{q}^{p}=UV^{\prime }$$

Once the rotation matrix describing the orientation between the LiDAR and camera data is obtained, it can be used as the initial input to a gradient descent optimization function. This iterative method refines both the rotation matrix and the translation (scale) vector by minimizing a defined cost function, thereby improving the overall calibration accuracy. Upon convergence, the optimized parameters are used to update the transformation tree between the LiDAR and camera frames, resulting in a more precise estimate of their relative pose.

The designed gradient descent state is presented as

(18) $$\theta =[p_{x_{i}}\;,p_{y_{i}},\;s,\;radMajb]$$

Here, $p_{x_{i}}$ and $p_{y_{i}}$ represent the LiDAR data points, which can be substituted with camera data as needed. The parameter s denotes the scale factor between the LiDAR and camera data, which is initially set to 1. The major rotation angle, radMajb, is computed using the singular value decomposition (SVD) result as follows:

(19) $$radMajb=atan2(V^{\prime }(2,1)V^{\prime }(1,1))$$

where $V^{\prime }$ is the right singular matrix obtained from the decomposition of the correlation matrix between the LiDAR and camera point sets.

The gradient descent method is a first-order iterative optimization algorithm used to minimize a cost function J(θ) with respect to its parameters θ. At each iteration, the parameters are updated in the opposite direction of the gradient of the cost function, aiming to find the local minimum. The parameter update rule is given by

(20) θ_new=θ_old−γ∇J(θ)

where θ the parameter vector to be optimized (e.g., rotation angle, scale factor, and translation), γ is the learning rate or step size (γ>0), ∇J(θ) is the gradient of the cost function with respect to θ, and J(θ) is the cost function, typically defined as the mean squared error between transformed LiDAR data and the corresponding camera data.

The gradient of the cost function J(θ), denoted as ∇J(θ) is derived to guide the optimization process in the gradient descent algorithm. It is expressed as follows:

(21) $$\nabla J(\theta )=[2q_{1}\left(p_{x_{i}}-{\bar{p}}_{x_{i}}\right),\;2q_{2}\left(p_{y_{i}}-{\bar{p}}_{y_{i}}\right),-2q_{3}\left(radMaja-s\times radMaja\right)\times s,\;-2q_{4}(radMaja-radMajb)]$$

where $p_{x_{i}}$ and $p_{y_{i}}$ are the LiDAR data coordinates for the i-th point, ${\bar{p}}_{x_{i}}$ and ${\bar{p}}_{y_{i}}$ are the corresponding mean values of the LiDAR data, s is the scale factor between the LiDAR and camera data (initially s=1), radMaja and radMajb represent major rotation angles extracted from SVD-based pattern alignment, $q=[q_{1},\;q_{2},\;q_{3},\;q_{4}]$ is a tuning matrix (or adjustment vector) used to scale the gradient components individually.

## 3. Experimental Results

### 3.1. Test Scenarios

To evaluate the robustness and performance of the proposed SVD-GD pattern matching algorithm, controlled misalignments were manually introduced into the system by modifying the extrinsic calibration parameters. These included rotational offsets, translation shifts, scale variations, and camera distortions, simulating practical calibration drift scenarios that may occur during long-term deployment in autonomous systems. The algorithm was tested across five representative cases. All experimental figures in this section were generated using MATLAB R2023a.

#### 3.1.1. General Misalignment Correction (Figure 6)

In this general case, random misalignments were introduced to simulate minor calibration drift. The algorithm iteratively refined rotation, translation, and scale parameters to minimize geometric discrepancies between LiDAR and camera detections. The blue outlines represent the initial LiDAR projections, the red outlines show CNN-based camera object detections, and the pink contours illustrate intermediate stages during optimization, demonstrating convergence toward accurate alignment.

**Figure 6:**
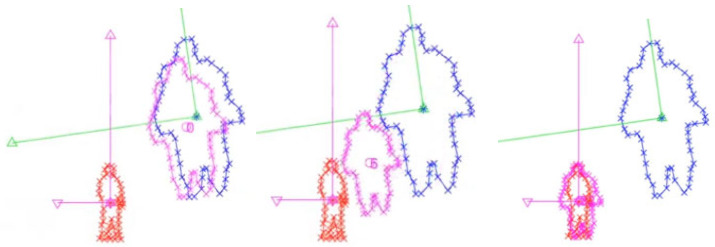
Iterative correction process of the SVD-GD algorithm. Blue: initial LiDAR projection; red: camera-based object detection; pink: intermediate results during optimization. The green and purple coordinate axes visualize the camera and LiDAR coordinate frames, respectively.

#### 3.1.2. Checkerboard Misalignment (Figure 7)

A controlled checkerboard target with known rotational and translational offsets was used to simulate sensor misalignment. The algorithm successfully corrected these errors, aligning the LiDAR projection with the camera-detected features, despite initial calibration offsets.

**Figure 7:**
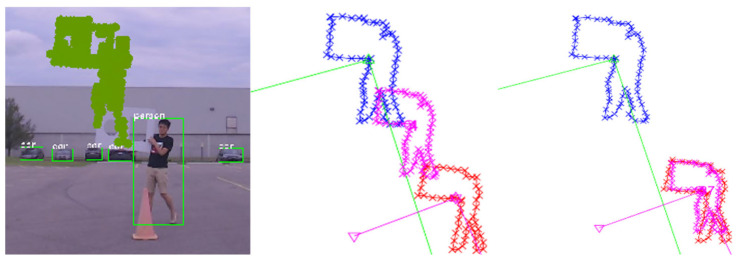
Correction under checkerboard misalignment scenario. Blue: initial LiDAR projection; red: camera detection; pink: intermediate results during optimization.

#### 3.1.3. Camera Distortion (Figure 8)

In this scenario, simulated camera lens distortion introduced additional projection errors. The algorithm effectively compensated for both geometric and projection misalignments by adjusting rotation, translation, and scale concurrently.

**Figure 8:**
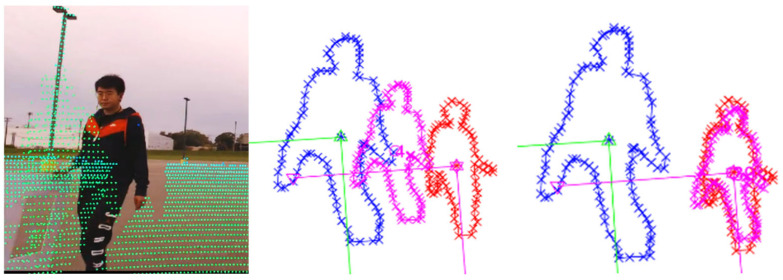
Correction under camera distortion scenario. Blue: initial LiDAR projection; red: camera detection; pink: intermediate results during optimization.

#### 3.1.4. LiDAR Mounting Drift (Figure 9)

To simulate mechanical mounting shifts, positional offsets were applied to the LiDAR sensor. The algorithm successfully recovered the correct transformation despite significant positional drifts between the two modalities.

**Figure 9:**
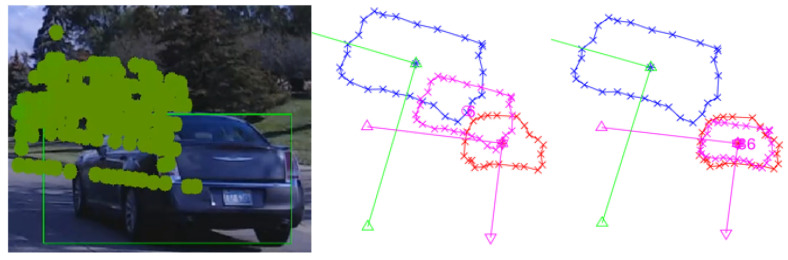
Correction under LiDAR mounting drift scenario. Blue: initial LiDAR projection; red: camera detection; pink: intermediate results during optimization.

#### 3.1.5. Extreme Misalignment (Figure 10)

In this most challenging case, large rotational, translational, and scaling errors were introduced, including severe orientation shifts. The algorithm demonstrated strong resilience by converging to the correct transformation parameters even under extreme calibration errors.

**Figure 10:**
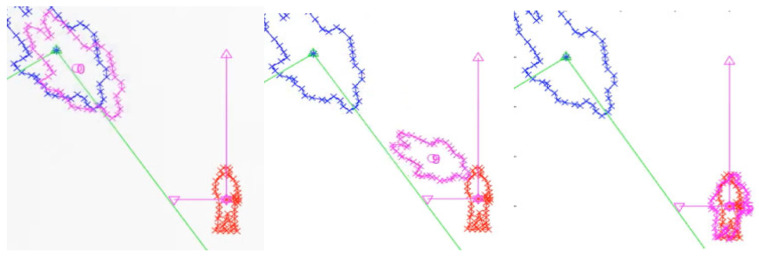
Correction under extreme misalignment scenario. Blue: initial LiDAR projection; red: camera detection; pink: intermediate results during optimization.

### 3.2. Quantitative Evaluation

To quantitatively evaluate the effectiveness of the proposed SVD-GD pattern matching algorithm, we measured the alignment accuracy across several real-world scenarios, including calibration drift, camera distortion, and extreme sensor misalignment. The metric used is the average 2D pixel distance or angular deviation between the projected LiDAR data and corresponding camera features, both before and after optimization. Additionally, the runtime per frame was recorded to assess computational efficiency. Table 1 summarizes the improvement in alignment accuracy and runtime performance for each test case.

## 4. Discussion

While the proposed SVD-GD pattern matching algorithm demonstrates strong alignment accuracy across multiple misalignment scenarios, several factors influencing sensor misalignment remain. Even though LiDAR sensors provide accurate depth measurements, practical deployment introduces relative errors between LiDAR and camera sensors due to physical installation tolerances, mounting vibrations, mechanical deformations, and thermal expansion during long-term operation. These factors result in a gradual drift of the extrinsic parameters, which can degrade fusion performance over time.

The current method relies on the availability of clear geometric features such as object contours, making it most effective when distinct objects, such as pedestrians or vehicles, are visible in the scene. Performance may degrade under severe occlusions, in highly cluttered environments, or when object detections are ambiguous. In addition, while the algorithm operates efficiently for single-object alignment (~0.4 s per frame), further optimization will be required for multi-object scenarios or real-time processing under high frame rates. Despite these challenges, the method provides a practical and adaptive solution for correcting misalignment in dynamic environments where traditional manual calibration is impractical. Despite these challenges, the proposed method offers a practical and adaptive solution for real-world autonomous systems, where continuous calibration is difficult to maintain

## 5. Conclusions and Future Work

This paper presented a pattern matching algorithm that maintains alignment between LiDAR and camera sensors by extracting geometric features and applying SVD-based transformation estimation with GD refinement. The proposed targetless approach was validated across multiple real-world scenarios, including sensor drift, camera distortion, and extreme misalignments, achieving alignment improvements exceeding 85% with final alignment errors reduced to sub-pixel and sub-degree levels.

By enabling continuous correction of calibration drift, the algorithm reduces dependence on repeated manual recalibration, offering long-term stability for autonomous systems operating in changing environments. Future work will focus on extending the method to handle multiple objects, improving robustness under occlusions, and integrating real-time adaptive calibration frameworks for autonomous driving applications.

## Figures and Tables

**Table 1:** Alignment accuracy improvement and runtime performance across test scenarios.

Scenario	Initial Misalignment	Residual Error After SVD-GD	Improvement (%)	Runtime per Frame (s)
Checkerboard Scene (Figure 7)	5.3 px	0.8 px	84.9%	0.34
Camera Distortion (Figure 8)	7.2 px	1.1 px	84.7%	0.39
LiDAR Drift on Highway (Figure 9)	10.0° rotation	1.2° rotation	88.0%	0.42
Extreme Misalignment (Figure 10)	45° + scale shift	2.5°/3.5 px	93.5% (avg)	0.47

## Data Availability

The data presented in this study is available on request from the corresponding author.

## Abbreviations

The following abbreviations are used in this manuscript:

LiDAR	Light Detection and Ranging
CNN	Convolutional Neural Network
SVD	Singular Value Decomposition
GD	Gradient Descent
ROS	Robot Operating System
PCD	Point Cloud Data
YOLO	You Only Look Once (object detection model)
